# Association of dietary niacin intake with all-cause and cardiovascular mortality of adult patients with chronic kidney disease in the United States: results from NHANES 1999–2018

**DOI:** 10.3389/fnut.2024.1436836

**Published:** 2024-08-30

**Authors:** Chao Zhang, Qi Cheng, Xinjun Yang, Wei Zhao, Kaifa Luo, Yunlong Qin

**Affiliations:** ^1^Department of Nephrology, Bethune International Peace Hospital, Shijiazhuang, Hebei, China; ^2^Outpatient Department, Bethune International Peace Hospital, Shijiazhuang, Hebei, China

**Keywords:** niacin, chronic kidney disease, mortality, national health and nutrition examination survey, cohort study

## Abstract

**Objective:**

The relationship between dietary niacin intake (DNI) and mortality rates among patients afflicted with chronic kidney disease (CKD) is a subject of debate. Utilizing data derived from the National Health and Nutrition Examination Survey (NHANES), this study adopts a retrospective cohort design with an aim to investigate the association in the American adult patients with CKD.

**Methods:**

A cohort study was conducted in the National Health and Nutrition Examination Survey (NHANES) between 2009 and 2018 that enrolled 6,191 CKD patients aged 20 years and above. We collected data on mortality through 31 December 2018. DNI was measured using a 24-h recall method. The relationship between DNI levels and mortality from all causes and cardiovascular causes was analyzed using weighted Cox proportional hazards models. The Kaplan–Meier (K-M) survival curve was plotted to illustrate these associations.

**Results:**

Following a median monitoring period of 85 months, it was observed that 2,419 individuals (33.08%) succumbed to all causes, whereas cardiovascular-related deaths were recorded for 746 participants (10.45%). When controlling for confounders, an inverse relationship was established between DNI and mortality rates. Specifically, a marginal increase of 1 mg/day in DNI corresponded to a reduced Hazard Ratios (HRs) of 0.993 (0.987, 0.999; *p* = 0.027) for all-cause mortality and 0.980 (0.969, 0.991; *p* < 0.001) for cardiovascular mortality. A further stratified analysis by quartiles of DNI, with the lowest quartile serving as the reference, revealed that the highest quartile was associated with HRs of 0.820 (0.697, 0.966) for all-cause mortality and 0.663 (0.465, 0.944) for cardiovascular mortality, both displaying a significant trend (*p* < 0.001). However, a subdivision of CKD patients by age showed that the protective effects of higher DNI were only confined to individuals aged 60 years and above but not to those under 60 years of age.

**Conclusion:**

A negative correlation between DNI and mortality due to all causes and cardiovascular issues among CKD patients aged 60 and above was revealed based on the datasets; however, this association was not observed in younger individuals under 60. Consequently, enhancing DNI might serve as a beneficial therapeutic strategy specifically for the older CKD demographic.

## 1 Introduction

Chronic kidney disease (CKD) is characterized by persistent structural or functional anomalies of the kidneys, lasting over 3 months, inclusive of indicators of kidney damage or a reduction in renal filtration capacity (1). The prevalence of CKD ranges between 10 and 15% worldwide, with a year-on-year increment noted (2). This condition profoundly undermines the quality of life and elevates the risk of mortality from all causes as well as cardiovascular events. According to estimates by the World Health Organization (WHO), CKD is directly responsible for between 5 and 10 million deaths globally each year (3). In individuals with end-stage kidney disease (ESRD), cardiovascular complications remain the predominant cause of increased mortality, with the mortality risk from such diseases being over 20 times higher in CKD patients than in the general populace (4). CKD has become a serious public health issue, and therefore the search for measures to delay the progression of CKD and reduce mortality is one of the most concerned problems in the field of nephrology.

Niacin, also known as vitamin B3, is an essential nutrient obtained through dietary intake, where food sources include liver, meat, fish, and whole grains (5). Historically employed for pellagra management, niacin now demonstrates significant anti-inflammatory and antioxidant capabilities across a multitude of cell types and tissues, which plays a crucial role in addressing various pathologies such as diabetes mellitus (DM), obesity, coronary artery disease (CAD), and atherosclerosis (6). Research indicates potential benefits of niacin in CKD through its modulation of lipid metabolism abnormalities, reduction in phosphate levels, enhancement of endothelial function, and its anti-inflammatory and antioxidant attributes, thereby contributing to a decrease in all-cause and cardiovascular-related mortalities among CKD sufferers (7, 8). Nonetheless, findings concerning the effectiveness of niacin in CKD remain inconclusive (9, 10). We also note that these investigations predominantly focus on niacin supplements, with minimal emphasis on niacin obtained through dietary sources. Current research on the association between dietary niacin intake (DNI) and mortality rates in CKD patients is scant.

To investigate the association between DNI and mortality outcomes due to all causes as well as cardiovascular conditions, we conducted an extensive cohort analysis utilizing data from the National Health and Nutrition Examination Survey (NHANES) spanning from 1999 to 2018. This retrospective study was based on a large dataset of American adults.

## 2 Materials and methods

### 2.1 Data and sample sources

The NHANES is a series of studies conducted to evaluate the health and nutritional statuses across various demographics in the United States (11). Utilizing a stratified, multistage probabilistic sampling method, NHANES collects a comprehensive set of data, including demographic profiles, dietary information, physical examinations, and laboratory measurements, which are integral to research in epidemiology and health sciences. The National Center for Health Statistics (NCHS) ethical review board has granted approval for all study protocols related to NHANES, ensuring that informed consent was secured from every participant.

**Figure 1:**
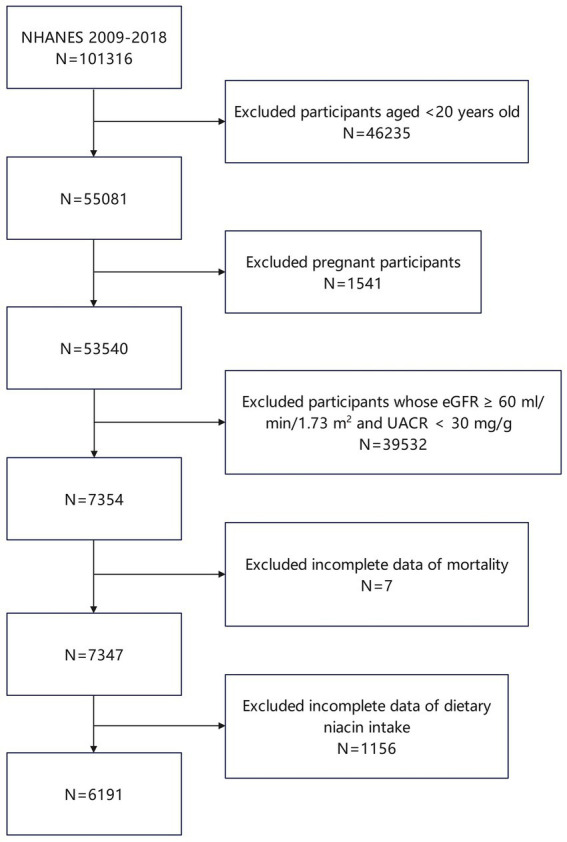
Flowchart of the participants selection from NHANES 1999–2018.

From 1999 to 2018, a total of 101,316 individuals have participated in 20 NHANES cycles (12). In the present study, we applied exclusion criteria to the participant pool as follows: individuals under 20 years of age were not included (totaling 46,235), as were those who were pregnant (1,541 excluded). Moreover, individuals demonstrating optimal renal function, defined by an estimated glomerular filtration rate (eGFR) of 60 mL/min/1.73 m^2^ or higher and a urine albumin-to-creatinine ratio (UACR) below 30 mg/g, were also excluded (39,532 individuals). Additionally, exclusions were made for participants lacking mortality data (7 individuals) and those with incomplete data on DNI (1,156 individuals). Following these exclusions, the study ultimately encompassed 6,191 participants (Figure 1).

### 2.2 Independent variable

DNI (mg/day) was primarily assessed using two 24-h dietary recalls, which were conducted to capture participants’ dietary intake, including both food and beverages, for the preceding 24-h period. The initial dietary recall was performed face-to-face in the Mobile Examination Center, ensuring accurate report under direct supervision. To enhance the accuracy of data, a second recall was conducted via telephone between 3 and 10 days after the initial session, allowing for the cross-verification of dietary intake. This recall approach helps to mitigate the variability of day-to-day food intake and reduces recall bias, thereby improving the reliability and validity of the dietary data. Trained data gatherers and nutritionists reviewed the data to ensure completeness and accuracy. Depending on the practicalities of data collection, in 1999–2002, dietary data from day 1 was used, and in 2003–2018, the average dietary data from 2 days was used. The exclusion criteria encompassed the absence of DNI among participants. The content of niacin in various foods and beverages was determined based on the U.S. Department of Agriculture’s Food and Nutrient Database for Dietary Studies (13).

### 2.3 Dependent variable

The estimation of eGFR was performed utilizing the creatinine equation developed by the CKD Epidemiology Collaboration in 2021 (14). CKD was identified when the eGFR fell below 60 mL/min/1.73 m^2^ or the UACR exceeded 30 mg/g (15). The study primarily investigated all-cause mortality, with cardiovascular mortality as a secondary consideration. The latter was specified as deaths attributed to cardiovascular disease, in accordance with the guidelines from the 10th revision of the International Statistical Classification of Diseases, Injuries, and Causes of Death (ICD-10) (15).

### 2.4 Covariates

Informed by prior research on niacin and CKD, the present study incorporated an array of covariates. Demographic variables such as sex, age, race, educational attainment, marital status, and the income-to-poverty ratio were considered. The presence of DM was established through a fasting blood glucose (FBG) level of 125 mg/dL or higher, self-reports of diabetes, or ongoing use of antihyperglycemic medications (16). Hypertension was identified when systolic blood pressure (SBP) averaged 140 mmHg or greater, or diastolic blood pressure (DBP) was 90 mmHg or higher, or if there was a physician’s diagnosis, or current use of antihypertensive drugs (17). The analysis categorized smoking status into never smokers (less than 100 cigarettes consumed in a lifetime), former smokers (100 or more cigarettes previously, but none currently), and active smokers (100 or more cigarettes with occasional or daily smoking) (18). Body Mass Index (BMI) was segmented into under 25, between 25 and 29.9, and 30 kg/m^2^ or above, denoting normal weight, overweight, and obesity, respectively (19). Self-reported health statuses ranged from poor to fair, to good, and then to very good or excellent. Covariates also included levels of alanine aminotransferase (ALT), aspartate aminotransferase (AST), and serum albumin (ALB). Data concerning cardiac diseases and cancer were extracted from self-administered questionnaires, with cardiac disease encompassing conditions such as heart failure, coronary heart disease, angina, or myocardial infarction.

### 2.5 Statistical analyses

According to the weight calculation method of NHANES, we used WTMEC4YR (1999–2002) and WTMEC2YR (2003–2018) to provide weights for all data. To assess baseline differences across various DNI levels, weighted t-tests and weighted chi-square tests were employed for continuous and categorical variables, respectively. Continuous variables were presented as mean values with standard deviations, while categorical data were shown as frequencies (percentages). To explore the association of DNI with both all-cause and cardiovascular mortality, Cox regression models were utilized, employing weights. Model 1 included no covariates to serve as a baseline comparison, allowing us to assess the unadjusted association between DNI and mortality. Model 2 was adjusted for a comprehensive set of demographic factors, including sex, age, race, educational attainment, marital status, and the income-to-poverty ratio. These variables were selected based on their established associations with both dietary intake and mortality outcomes in the literature. Model 3 included additional adjustments for health-related variables such as smoking status, BMI, self-reported health status, DM, hypertension, cardiac disease, cancer, and key biochemical indexes including ALT, AST, ALB. The selection of these covariates was based on their documented impact on mortality in CKD populations, as well as their potential to confound the relationship between DNI and mortality. A detailed list of covariables is shown in Supplementary Table S2. Considering the potential impact of age on DNI’s effects, participants were categorized into two groups using a threshold age of 60 years for additional analyses. The findings were quantified as hazard ratios (HRs) with 95% confidence intervals (95%CI). Interaction terms were analyzed to determine variability in the relationships across different subgroups. All statistical analyses were performed using R software, version 4.3.2. Statistical significance was determined using a *p*-value threshold of 0.05. All analyses where the *p*-value was below this threshold were considered statistically significant.

## 3 Results

### 3.1 Baseline characteristics of participants

The study incorporated 6,191 subjects, correlating to a demographic scale of 20 million based on weighting calculations. Participants had an average age of 58.87 ± 17.94 years and consisted of 49.21% males and 50.79% females. DNI averaged 21.91 ± 11.19 mg/day. Throughout a median monitoring period of 85 months (interquartile range: 44, 140), all-cause mortality was recorded for 2,419 subjects (33.08%), and cardiovascular mortality was noted in 746 subjects (10.45%). Participants were divided into quartiles based on their DNI levels. The specific ranges for each quartile were as follows:

Quartile 1 (Q1): DNI < 14.4 mg/day.

Quartile 2 (Q2): 14.4 mg/day ≤ DNI < 19.7 mg/day.

Quartile 3 (Q3): 19.7 mg/day ≤ DNI < 26.9 mg/day.

Quartile 4 (Q4): DNI ≥ 26.9 mg/day.

These ranges were determined based on the distribution of DNI values among the participants and were used to explore the potential dose–response relationship between DNI and mortality outcomes. Baseline characteristics of the participants across these quartiles are detailed in Table 1.

**Table 1:** Baseline characteristics of weighted sample by the quartiles of DNI.

	Overall	Q1 < 14.4 mg/day	Q2 14.4–19.7 mg/day	Q3 19.7–26.9 mg/day	Q4 ≥ 26.9 mg/day	*p*-value
	*N* = 6,191	*N* = 1,544	*N* = 1,540	*N* = 1,558	*N* = 1,549	
Sex (%)						< 0.001
Female	50.79	70.56	61.23	46.64	30.06	
Male	49.21	29.44	38.77	53.36	69.94	
Age (years)	58.87 ± 17.94	61.09 ± 18.58	61.70 ± 17.25	58.56 ± 17.78	54.93 ± 17.46	< 0.001
Race (%)						0.069
Mexican American	7.32	7.81	6.86	7.05	7.59	
Non-Hispanic Black	13.58	16.96	12.51	12.92	12.49	
Non-Hispanic White	67.78	62.73	70.27	68.6	68.74	
Other Hispanic	5.2	6.46	4.53	5.24	4.77	
Other Race	6.13	6.05	5.83	6.19	6.4	
Education (%)						< 0.001
College or more	49.99	39.36	47.31	53.81	57.12	
High school	25.56	25.01	28.21	23.68	25.38	
Middle school or lower	24.44	35.62	24.48	22.51	17.5	
Marital status (%)						< 0.001
Never married	11.13	9.51	9.06	11.38	13.99	
Married/with partner	59.91	51.88	58.43	62.47	65.09	
Widowed/divorced/separated	28.96	38.61	32.51	26.16	20.92	
Income-to-poverty ratio (%)						< 0.001
<1.3	26.24	36.44	26.21	22.47	21.7	
≥1.3	73.76	63.56	73.79	77.53	78.3	
Smoke status (%)						0.125
never smokers	48.2	50.6	49.5	47.62	45.72	
ever smokers	33.41	29.48	34.27	34.22	34.97	
current smokers	18.4	19.93	16.24	18.16	19.32	
BMI (%)						0.058
<25	25.6	26.7	26.52	26.31	23.32	
25–30	32.13	35.88	30.52	30.63	32.05	
≥30	42.27	37.43	42.96	43.07	44.63	
Health Status (%)						0.004
Poor to Fair	30.05	36.31	29.77	29.22	26.61	
Good	39.09	37.13	39.51	40.51	38.8	
Very good to Excellent	30.86	26.56	30.72	30.27	34.59	
Hypertension (%)						0.035
Yes	63.36	64.74	66.58	63.29	59.53	
No	36.64	35.26	33.42	36.71	40.47	
DM (%)						0.181
Yes	33.93	32.78	34.25	36.69	32.03	
No	66.07	67.22	65.75	63.31	67.97	
Cardiac disease (%)						0.024
Yes	8.73	10.47	9.1	8.69	7.1	
No	91.27	89.53	90.9	91.31	92.9	
Cancer (%)						0.281
Yes	16.56	17.15	17.95	16.76	14.69	
No	83.44	82.85	82.05	83.24	85.31	
All-cause mortality (%)	33.08	41.89	36.62	30.46	25.52	< 0.001
Cardiovascular mortality (%)	10.45	13.62	11.72	9.89	7.39	< 0.001

Statistical analyses revealed significant disparities in several demographic and health variables across quartiles of DNI. These variables include sex, age, educational attainment, marital status, the ratio of income to poverty, presence of hypertension, self-reported health statuses, incidence of cardiac conditions, and mortality due to all causes and cardiovascular issues (*p* < 0.05). Conversely, no significant differences were observed among the quartiles in terms of race, smoking habits, BMI, DM, and cancer prevalence (*p* ≥ 0.05). Individuals classified Q1 group (i.e., lower DNI) were predominantly female, of advanced age, with education extending to middle school or lower. This group also included a higher proportion of widowed, divorced, or separated individuals, a lower income-to-poverty ratio, and more frequent reports of fair to poor health. Moreover, these individuals exhibited a higher prevalence of hypertension and cardiac disorders, correlating with increased mortality rates from all causes and cardiovascular diseases compared to those in higher DNI quartiles.

### 3.2 Relationship between DNI and mortality

**Figure 2:**
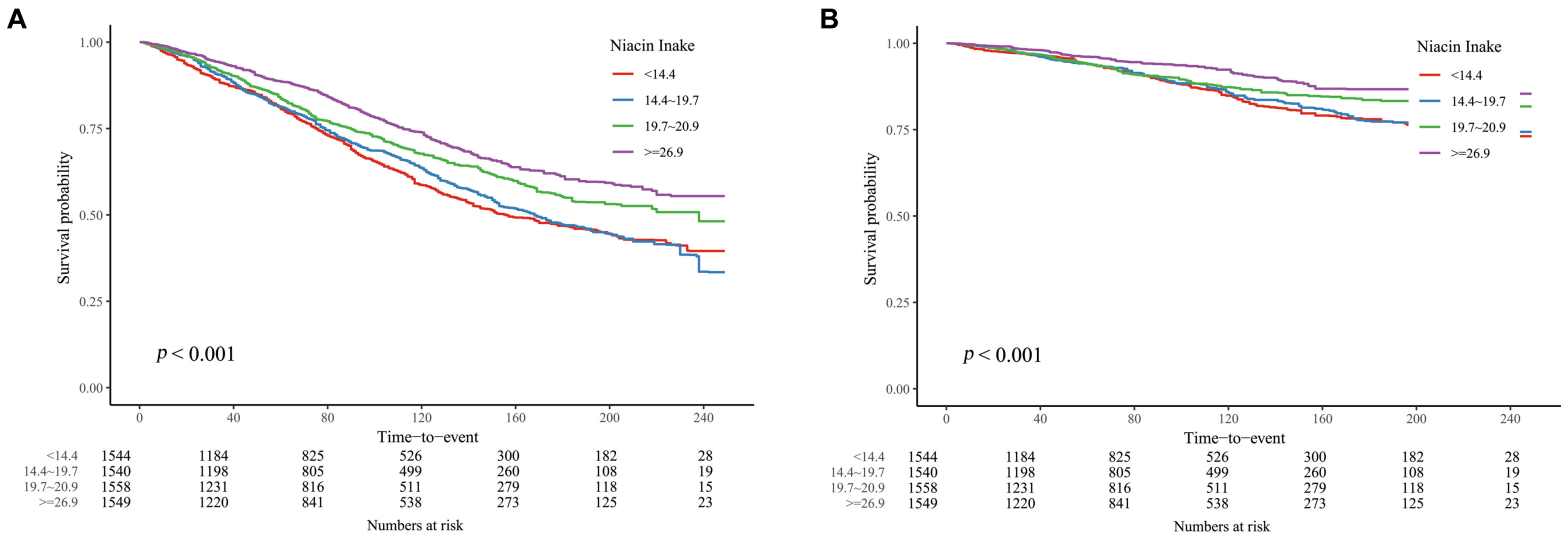
K-M curves describing the relationship between DNI and mortality in CKD patients. **(A)** all-cause mortality **(B)** cardiovascular mortality.

We conducted weighted Cox regression analyses to examine the relationship between DNI and mortality outcomes due to all causes and cardiovascular diseases. Model 1 involved no covariate adjustments. Model 2 included adjustments for demographic and socio-economic factors such as age, sex, race, marital status, education, and the income-to-poverty ratio. Model 3 encompassed adjustments for all previously mentioned covariates. Due to the direct correlation between cardiac disease and cardiovascular deaths, cardiac disease was excluded as a covariate in the cardiovascular mortality analysis within Model 3. Statistically significant relationships were found between DNI and mortality from all causes as well as cardiovascular conditions across all models (*p* < 0.01; Tables 2, 3). Specifically in Model 3, for each 1 mg/day increment in DNI, the HRs were 0.993 (0.987–0.999) for all-cause mortality and 0.980 (0.969–0.991) for cardiovascular mortality. Additionally, when examining the association of DNI across quartiles with mortality outcomes (with Q1 serving as the reference), the HRs for Q4 were 0.820 (0.697–0.966) for all-cause mortality and 0.663 (0.465–0.944) for cardiovascular mortality. The significant *p* for trend values (*p* < 0.001 for both all-cause and cardiovascular mortality) indicate a consistent and statistically significant decrease in mortality risk with increasing DNI levels across the quartiles. This suggested a dose–response relationship, where higher DNI intake was associated with progressively lower risks of mortality. These HR values suggested a modest but statistically significant protective effect of higher DNI against mortality. Clinically, this implied that even a small increase in dietary niacin intake could contribute to a reduction in the risk of death, particularly from cardiovascular causes. For example, the HR of 0.980 for cardiovascular mortality indicated that each additional 1 mg/day of DNI was associated with a 2% reduction in the risk of cardiovascular death. Although this reduction may appear small on an individual level, it could translate into substantial public health benefits when considered across a larger population, especially in high-risk groups such as CKD patients. The consistent protective trend observed across different models and quartiles underscored the potential of DNI as a modifiable dietary factor in improving long-term outcomes in CKD patients. K-M curves, stratified by quartiles of DNI, were displayed in Figure 2. The curve representing the Q1 showed the lowest survival probability for all-cause mortality, with a statistically significant difference (log-rank test, *p* < 0.001). A similar trend was noted for cardiovascular mortality. These results underscored the inverse relationship between DNI levels and mortality rates in patients with CKD.

**Table 2:** The association between DNI and all-cause mortality in CKD patients, weighted.

	OR (95% CI)		
All participants	Model 1	Model 2	Model 3
DNI (mg/day)	0.982 (0.976,0.987)	0.990 (0.985,0.996)	0.993 (0.987,0.999)
*p* value	< 0.001	0.001	0.027
DNI quartiles			
Q1, < 14.4 mg/day	Ref	Ref	Ref
Q2, 14.4–19.7 mg/day	0.952 (0.810,1.120)	0.936 (0.803,1.092)	0.983 (0.845,1.143)
Q3, 19.7–26.9 mg/day	0.769 (0.654,0.904)	0.843 (0.714,0.995)	0.839 (0.712,0.990)
Q4, ≥ 26.9 mg/day	0.613 (0.522,0.719)	0.764 (0.652,0.895)	0.820 (0.697,0.966)
*p* for trend	< 0.001	< 0.001	0.005
Participants ≥ 60 years old			
DNI (mg/day)	0.986 (0.981,0.991)	0.985 (0.979,0.992)	0.990 (0.983,0.996)
*p* value	< 0.001	< 0.001	0.002
DNI quartiles			
Q1, < 13.8 mg/day	Ref	Ref	Ref
Q2, 13.8–18.7 mg/day	0.880 (0.761,1.019)	0.906 (0.765,1.072)	0.919 (0.779,1.085)
Q3, 18.7–25.2 mg/day	0.783 (0.670,0.915)	0.764 (0.628,0.928)	0.804 (0.674,0.958)
Q4, ≥ 25.2 mg/day	0.715 (0.620,0.824)	0.717 (0.603,0.851)	0.789 (0.656,0.950)
*p* for trend	< 0.001	< 0.001	0.005
Participants < 60 years old			
DNI (mg/day)	1.003 (0.992,1.015)	1.002 (0.987,1.017)	1.001 (0.986, 1.016)
*p* value	0.580	0.800	0.886
DNI quartiles			
Q1, < 16.11 mg/day	Ref	Ref	Ref
Q2, 16.11–21.95 mg/day	1.194 (0.755,1.89)	1.123 (0.588,2.112)	0.981 (0.606,1.589)
Q3, 21.95–30.48 mg/day	0.953 (0.632,1.44)	0.797 (0.575,1.808)	0.801 (0.485,1.322)
Q4, ≥ 30.48 mg/day	1.144 (0.764,1.71)	0.975 (0.608,1.647)	0.917 (0.583,1.440)
*p* for trend	0.748	0.651	0.588

**Table 3:** The association between DNI and cardiovascular mortality in CKD patients, weighted.

	OR (95% CI)		
All participants	Model 1	Model 2	Model 3
DNI (mg/day)	0.973 (0.964,0.982)	0.981 (0.971,0.990)	0.980 (0.969,0.991)
*p* value	< 0.001	< 0.001	< 0.001
DNI quartiles			
Q1, < 14.4 mg/day	Ref	Ref	Ref
Q2, 14.4–19.7 mg/day	0.935 (0.738,1.186)	0.921 (0.714,1.189)	0.966 (0.717,1.301)
Q3, 19.7–26.9 mg/day	0.765 (0.590,0.993)	0.861 (0.660,1.124)	0.821 (0.635,1.062)
Q4, ≥ 26.9 mg/day	0.544 (0.413,0.718)	0.673 (0.499,0.907)	0.663 (0.465,0.944)
*p* for trend	< 0.001	0.006	0.007
Participants ≥ 60 years old			
DNI (mg/day)	0.979 (0.968,0.990)	0.978 (0.967,0.990)	0.977 (0.964,0.991)
*p* value	< 0.001	< 0.001	0.001
DNI quartiles			
Q1, < 13.8 mg/day	Ref	Ref	Ref
Q2, 13.8–18.7 mg/day	0.900 (0.716,1.131)	0.891 (0.684,1.160)	0.984 (0.719,1.347)
Q3, 18.7–25.2 mg/day	0.812 (0.646,1.019)	0.816 (0.613,1.085)	0.866 (0.633,1.185)
Q4, ≥ 25.2 mg/day	0.673 (0.509,0.891)	0.666 (0.490,0.904)	0.697 (0.492,0.987)
*p* for trend	0.002	0.006	0.021
Participants < 60 years old			
DNI (mg/day)	0.993 (0.973,1.013)	0.985 (0.960,1.010)	0.984 (0.958,1.011)
*p* value	0.500	0.226	0.234
DNI quartiles			
Q1, < 16.11 mg/day	Ref	Ref	Ref
Q2, 16.11–21.95 mg/day	1.333 (0.590,3.011)	1.512 (0.642,3.561)	1.243 (0.566,2.727)
Q3, 21.95–30.48 mg/day	1.224 (0.628,2.386)	1.125 (0.513,2.465)	1.155 (0.436,3.062)
Q4, ≥ 30.48 mg/day	0.960 (0.382,2.412)	0.798 (0.270,2.359)	0.833 (0.240,2.898)
*p* for trend	0.873	0.507	0.734

**Figure 3:**
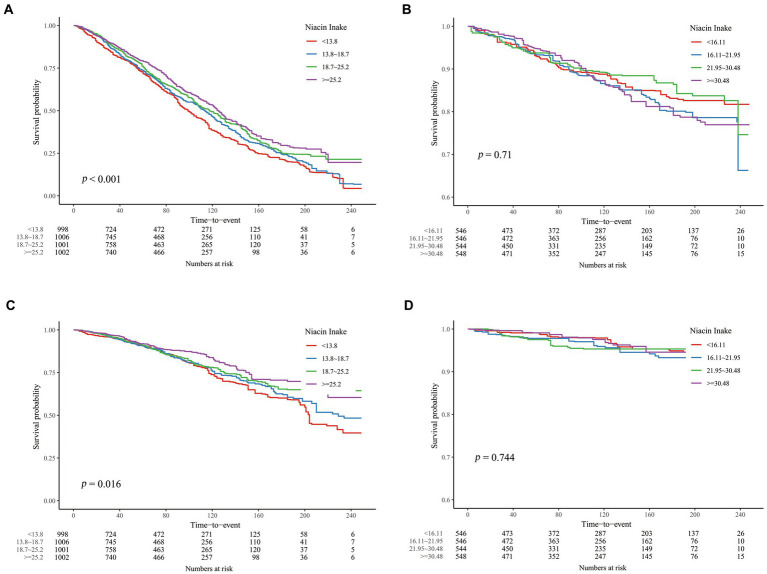
K-M curves describing the relationship between DNI and mortality in CKD patients of different age stages. **(A)** all-cause mortality, ≥ 60 years old; **(B)** all-cause mortality, < 60 years old; **(C)** cardiovascular mortality, ≥ 60 years old; **(D)** all-cause mortality, < 60 years old.

However, in the analysis of CKD patients at different ages, we found that the associations between DNI and mortality were distinctly different above and under 60 years of age (Tables 2, 3). In the age group ≥60 years, weighted COX regression analysis demonstrated a consistent inverse relationship between DNI and mortality due to all causes and cardiovascular events across Models 1 to 3 (*p* < 0.01). Specifically, in Model 3, the risk of mortality from all causes was reduced by 21.1%, and cardiovascular mortality was reduced by 30.1% in the Q4 compared to the Q1, with a significant trend (*p* < 0.05). Conversely, for participants below 60, the analyses across Models 1 to 3 revealed no significant association between DNI or its quartile divisions and mortality rates from either all causes or cardiovascular issues (*p* ≥ 0.05). The K-M curves for these two groups similarly reflected this difference (Figure 3).

### 3.3 Subgroup analysis

**Figure 4:**
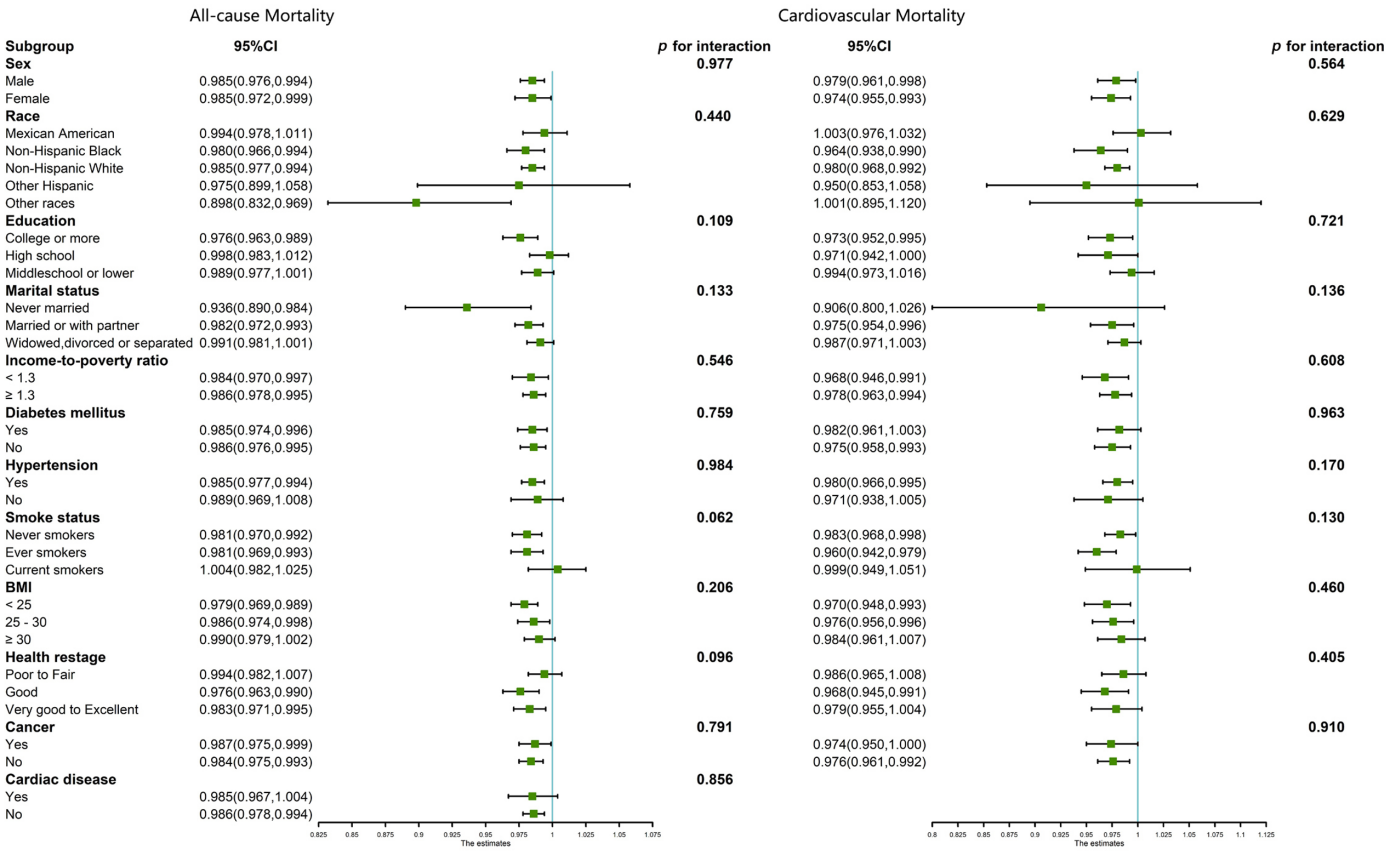
Subgroup analysis for the association between DNI and all-cause and cardiovascular mortality in CKD patients ≥60 years of age, weighted.

Subsequent subgroup analysis targeted individuals aged 60 years or older, with the data depicted in forest plots (Figure 4). These subgroups underwent stratification based on various demographic and health-related factors, including sex, race, educational level, marital status, income-to-poverty ratio, smoking habits, BMI, self-reported health statuses, DM, cancer, and cardiac disease (only in all-cause mortality). The investigations revealed a consistently negative correlation between DNI and mortality rates—both all-cause and cardiovascular—across all stratifications among patients with CKD who were 60 years of age or older (*p* > 0.05 for all interactions).

## 4 Discussion

Niacin has been recognized as the earliest therapeutic agent for dyslipidemia management and is effective in mitigating the risk of atherosclerotic cardiovascular diseases (20). Currently, the data on niacin’s impact on CKD present mixed outcomes. Some researches indicate the potential of niacin to mitigate several adverse factors affecting renal function, such as hyperlipidemia, oxidative stress, inflammation, and endothelial dysfunction (21). These benefits contribute to the deceleration of the decline in glomerular filtration rate, reduction in cardiovascular risks, and overall enhancement of CKD outcomes. Additionally, niacin may decrease serum phosphorus by inhibiting the gastrointestinal absorption of dietary phosphorus, thus ameliorating mineral and bone disorders in ESRD patients (22). Contrarily, other investigations have found niacin to be ineffective in improving CKD. A multicenter prospective study in the United States revealed that, although niacin combined with simvastatin improved triglycerides (TG) and high-density lipoprotein (HDL) levels in CKD patients, it did not enhance cardiovascular or renal outcomes and was linked to increased all-cause mortality (10). Tilman, a researcher from France, has argued the lack of sufficient evidence supporting the survival benefit of niacin in regulating serum phosphate in CKD patients (9). Furthermore, current researches on niacin and CKD mainly focus on niacin supplements, and no large-scale studies specifically addressing the relationship between DNI and CKD have been retrieved. In light of the above, utilizing the NHANES database, we conducted a comprehensive retrospective cohort investigation to delve into the association between DNI and mortality rates among CKD patients.

The present study encompassed 6,191 subjects, each showing an average DNI of 21.91 ± 11.19 mg/day. Recorded rates of all-cause and cardiovascular mortality stood at 33.08 and 10.45%, respectively. Employing weighted Cox regression analyses, we observed a significant inverse association between higher DNI levels and reduced mortality due to all causes and cardiovascular events in patients suffering from CKD, after adjusting for pertinent confounders. However, subgroup analysis by age revealed that this inverse relationship was evident exclusively in individuals aged 60 or above, whereas it was not detectable in those under 60. Thus, the results of this study supported a beneficial effect of dietary niacin on the prognosis of CKD, but this effect was only present in patients aged 60 and above. The age-specific protective effect of higher DNI observed in individuals aged 60 and above could be attributed to several biological and lifestyle factors. First, as people age, metabolism also changes, including alterations in lipid metabolism, where niacin is known to influence positively. Second, older individuals may also experience a decline in renal function and an increase in oxidative stress and inflammation, conditions that niacin’s antioxidant and anti-inflammatory properties could help mitigate. Third, the prevalence of cardiovascular diseases increases with age, and the role of niacin in improving lipid profiles and reducing atherosclerotic risk could be more pronounced in this demographic. Fourth, older individuals often have different dietary patterns and nutrient absorption rates compared to younger individuals, potentially making them more responsive to the effects of niacin. These factors combined suggest that the elderly may particularly benefit from higher DNI as a protective measure against mortality, which aligns with our findings. It is also important to recognize that younger patients with CKD generally face a lower risk of mortality, a factor that could mask the potential effects of DNI. In this study, the small sample size of CKD patients under 60 years may have limited the statistical power needed to identify a significant association. This underscores the necessity for larger sample sizes and further research to investigate nutritional interventions that could be particularly relevant to younger CKD patients.

Based on previous studies, we collected four factors related to niacin and CKD from NHANES: TG, HDL, serum phosphorus, and systemic immunoinflammatory index (SII), and looked at their distributions in different DNI groups. High TG, low HDL and high blood phosphorus are thought to have a detrimental effect on the progression and prognosis of CKD (23–25). SII is a new index of systemic inflammation, and some studies have confirmed that it is positively associated with mortality in CKD patients (26). Among CKD patients aged 60 or older, grouped by quartiles of DNI, the distributions of the above four indicators were shown in Supplementary Table S1. TG, HDL and serum phosphorus were statistically different between the four groups, while SII was not. However, serum phosphorus, although decreasing with increasing DNI, did not show large gaps between the means of the four groups (Q1 3.71 mg/dL vs. Q4 3.63 mg/dL), which did not seem to be sufficient to have a significant effect on CKD mortality from clinical experience. TG and HDL, although significantly different between the four groups, tended to change in the opposite direction than expected: there was a tendency for TG to increase (Q1 165.88 mg/dL vs. Q4 176.57 mg/dL) and HDL to decrease (Q1 1.40 mmol/L vs. Q4 1.29 mmol/L) as DNI increased, which in turn may have had a detrimental effect on CKD. Consequently, the findings of our investigation implied that the influences of DNI on mortality among CKD patients might not be mediated through enhancements in TG, HDL, serum phosphorus, or inflammation levels. Further research is called for to elucidate the underlying mechanisms.

According to the US Institute of Medicine, the established daily niacin intake recommendation is 16 milligrams per day for adult males and 14 milligrams per day for adult females, with the tolerable upper intake level set at 35 milligrams per day (27). In our study, the mean intake of dietary niacin of the group ≥60 years old was 20.5 mg/day, which was above average but still far from the upper limit. Also, the upper limit for niacin (35 mg/day) is based on excessive intake of niacin in the form of supplements and additives, and does not apply to niacin in foods (28). Adverse effects due to excessive intake of niacin from foods have not been reported. Therefore, it may be feasible to appropriately elevate DNI to lower the mortality of CKD patients ≥60 years old.

Although the 24-h dietary recall method was widely used for dietary assessment due to its practicality and ability to capture detailed intake data, it was not without limitations. A primary limitation was the reliance on participants’ memory, which could introduce recall bias. Participants may forget or misreport their food intake, leading to inaccuracies in the reported DNI. Additionally, the 24-h recall captured dietary intake for only one or two specific days, which might not represent usual intake, particularly in individuals with highly variable diets. This limitation could result in measurement errors, potentially attenuating the observed associations between DNI and mortality. To mitigate this, we only included participants whose dietary recall status was reliable and used the mean of two dietary recalls where data allowed, but some degree of measurement error might still exist. These limitations suggested that while our findings indicated a significant association between DNI and mortality, caution should be exercised in interpreting the results, and further studies using more comprehensive dietary assessment methods may be needed to confirm these findings. Another limitation of this study was that we did not conduct a gender-specific analysis to explore potential differences in the relationship between DNI and mortality across genders. Since our primary focus was on age-related differences, future research is needed to investigate whether gender plays a significant role in modifying the effects of DNI on mortality outcomes.

## 5 Conclusion

Research indicates that in CKD patients aged 60 years or older residing in the United States, there is a negative correlation between DNI and mortality rates due to all causes and cardiovascular issues. Conversely, this correlation is absent in CKD patients under the age of 60. It is suggested that enhancing DNI could serve as a beneficial intervention for older CKD patients.

## Data Availability

The datasets presented in this study can be found in online repositories. The names of the repository/repositories and accession number(s) can be found at: https://www.cdc.gov/nchs/nhanes/index.htm.

## Supplementary material

The Supplementary material for this article can be found online at:

https://www.frontiersin.org/articles/10.3389/fnut.2024.1436836/full#supplementary-material
